# Hydrazinium 2-amino-4-nitro­benzoate dihydrate: crystal structure and Hirshfeld surface analysis

**DOI:** 10.1107/S2056989017004352

**Published:** 2017-03-24

**Authors:** James L. Wardell, Mukesh M. Jotani, Edward R. T. Tiekink

**Affiliations:** a Fundaçaö Oswaldo Cruz, Instituto de Tecnologia em Fármacos-Far Manguinhos 21041-250 Rio de Janeiro, RJ, Brazil; b Department of Chemistry, University of Aberdeen, Old Aberdeen, AB24 3UE, Scotland; c Department of Physics, Bhavan’s Sheth R. A. College of Science, Ahmedabad, Gujarat 380 001, India; d Research Centre for Chemical Crystallography, School of Science and Technology, Sunway University, 47500 Bandar Sunway, Selangor Darul Ehsan, Malaysia

**Keywords:** crystal structure, carboxyl­ate, salt, hydrogen bonding, Hirshfeld surface analysis

## Abstract

In the anion of the title salt hydrate, H_5_N_2_^+^·C_7_H_5_N_2_O_4_^−^·2H_2_O, the carboxyl­ate and nitro groups lie out of the plane of the benzene ring to which they are bound [dihedral angles = 18.80 (10) and 8.04 (9)°, respectively], and as these groups are conrotatory, the dihedral angle between them is 26.73 (15)°. An intra­molecular amino-N—H⋯O(carboxyl­ate) hydrogen bond is noted. The main feature of the crystal packing is the formation of a supra­molecular chain along the *b* axis, with a zigzag topology, sustained by charge-assisted water-O—H⋯O(carboxyl­ate) hydrogen bonds and comprising alternating twelve-membered {⋯OCO⋯HOH}_2_ and eight-membered {⋯O⋯HOH}_2_ synthons. Each ammonium-N—H atom forms a charge-assisted hydrogen bond to a water mol­ecule and, in addition, one of these forms a hydrogen bond with a nitro-O atom. The amine-N—H atoms form hydrogen bonds to carboxyl­ate-O and water-O atoms, and the amine N atom accepts a hydrogen bond from an amino-H atom. The hydrogen bonds lead to a three-dimensional architecture. An analysis of the Hirshfeld surface highlights the major contribution of O⋯H/H⋯O hydrogen bonding to the overall surface, *i.e*. 46.8%, compared with H⋯H contacts (32.4%).

## Abstract 2

In the title salt dihydrate, the conrotatory relationship between the carboxyl­ate and nitro groups of the anion leads to a dihedral angle between them of 26.73 (15)°. Substantial charge-assisted water-O—H⋯O(carboxyl­ate) hydrogen bonding leads to supra­molecular zigzag chains. These are connected into a three-dimensional architecture by N—H⋯O and N—H⋯N hydrogen bonds.

## Chemical context   

The present structure determination of the title salt dihydrate, [NH_2_NH_3_][O_2_C_6_H_4_NO_2_-4]·2H_2_O (I), is a continuation of on-going structural studies of the relatively unexplored chemistry of 2-amino-4-nitro­benzoic acid. This acid carries several groups capable of hydrogen bonding, *viz*. carb­oxy­lic/carboxyl­ate, amino and even nitro, and is anti­cipated to form crystals with significant hydrogen-bonding inter­actions, in both its neutral and deprotonated forms. Beyond the structure determination of several polymorphs of the parent structure (Wardell & Tiekink, 2011 ▸; Wardell & Wardell, 2016 ▸) and its 1:1 co-crystal with bis­(pyridin-2-yl)methanone and 2:1 co-crystal with 2-amino-4-nitro­benzoic acid (Wardell & Tiekink, 2011 ▸), all other investigations have been of deprotonated forms of the acid. Thus, the anion has been found coordinating in the carboxyl­ate-O, amino-N mode towards Pb^II^ in the coordination polymer *catena*-[bis­(μ_2_-2-amino-4-nitro­benzoato)lead(II)] (Chen & Huang, 2009 ▸), with the remaining literature structures being salts. These are either alkali metal salts, *i.e*. Na^+^, K^+^ (Smith, 2013 ▸), Rb^+^ (Smith, 2014*a* ▸) and Cs^+^ (Smith & Wermuth, 2011 ▸), or are ammonium salts, as discussed below. Herein, the crystal and mol­ecular structures of (I) are described along with an evaluation of its Hirshfeld surface.
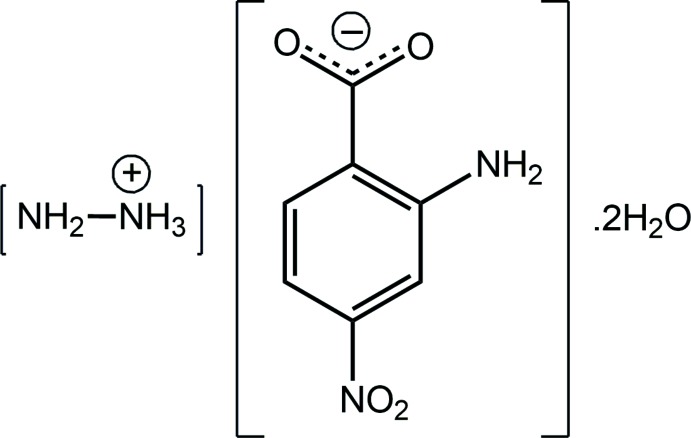


## Structural commentary   

**Figure 1:**
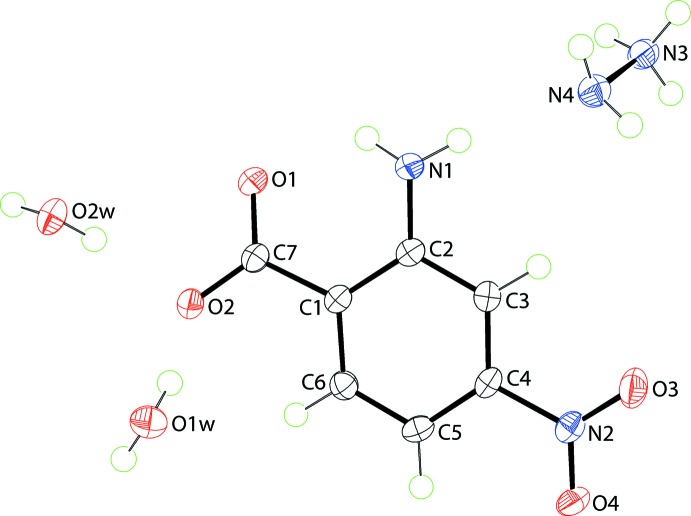
The mol­ecular structures of the asymmetric unit of (I), showing displacement ellipsoids at the 70% probability level.

The mol­ecular structures of the constituents of (I) are shown in Fig. 1 ▸; the asymmetric unit comprises one hydrazinium cation, one 2-amino-4-nitro­benzoate anion and two water mol­ecules of crystallization. In all-organic structures, when protonated in crystals, hydrazine is ten times more likely to be present as a mono-protonated hydrazinium cation rather than in its the diprotonated form, *i.e.* hydrazine-1,2-diium di-cation (Groom *et al.*, 2016 ▸); when non-organic structures are also considered, this ratio increases to 20:1. The confirmation of the mono-protonation in (I) is found in the pattern of inter­molecular inter­actions, in particular in the observation that the amine-N4 atom accepts a hydrogen bond (see below). The N3—N4 bond length in (I) is 1.4492 (15) Å. The assignment of deprotonation of 2-amino-4-nitro­benzoic acid during co-crystallization is readily adduced in the near equivalence of the carboxyl­ate C—O bond lengths, *i.e*. C7—O1 = 1.2579 (15) and C7—O2 = 1.2746 (15) Å. While there is an intra­molecular amino-N—H⋯O(carboxyl­ate) hydrogen bond, Table 1 ▸, a significant twist of the carboxyl­ate group with respect to the benzene ring to which it is connected is noted, as evidenced in the value of the C2—C1—C7—O1 torsion angle of 18.83 (17)°. With respect to the nitro group, this is also twisted but, to a lesser extent: the O3—N2—C4—C3 torsion angle is 7.53 (16)°. The terminal groups are conrotatory, forming a dihedral angle of 26.73 (15)°.

## Supra­molecular features   

**Figure 2:**
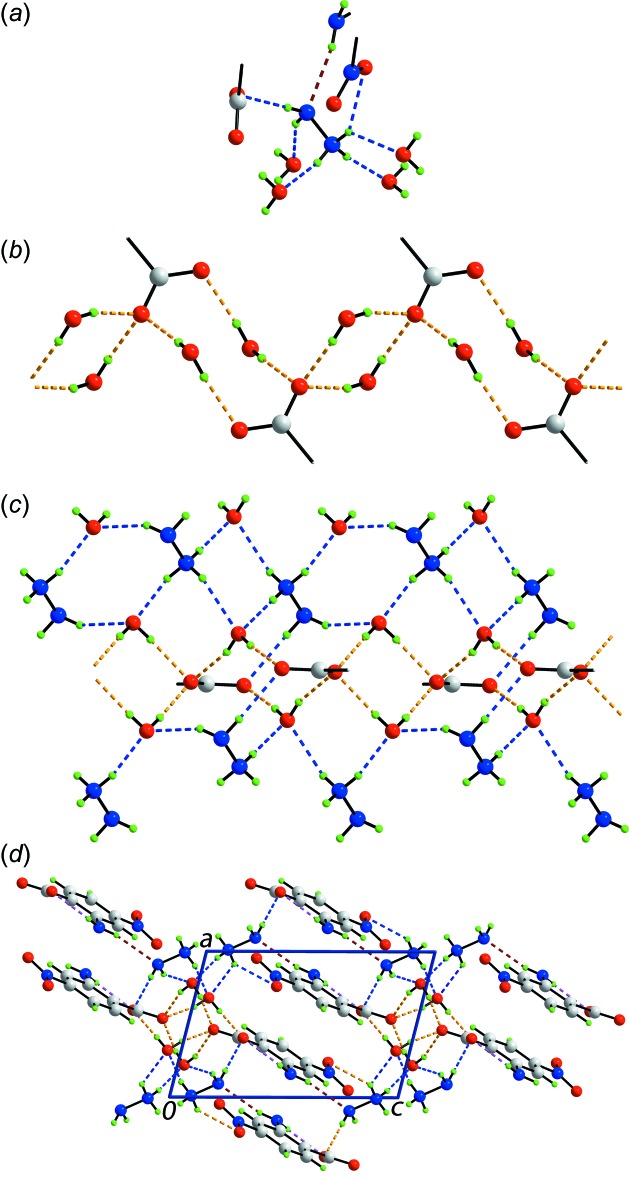
The mol­ecular packing in (I): (*a*) immediate environment about the [H_2_NNH_3_] cation, (*b*) supra­molecular chain comprising anions and water mol­ecules only, orientated along the *b* axis and sustained by water-O—H⋯O(carboxyl­ate) hydrogen bonding, (*c*) decoration of the chain of (*b*) with cations and additional water mol­ecules to highlight the formation of various supra­molecular synthons (see text) and (*d*) a view of the unit-cell contents in projection down the *b* axis. The O—H⋯O, N—H⋯O, N—H⋯N and intra­molecular N—H⋯O hydrogen bonds are shown as orange, blue, brown and pink dashed lines, respectively. In (*b*) and (*c*), all but the CO_2_ groups of the two central benzoate residues have been removed for clarity.

As expected from the chemical composition of (I), there are a number of conventional hydrogen-bonding inter­actions in the crystal, involving all possible hydrogen-bond donors and acceptors, Table 1 ▸. These sustain a three-dimensional architecture. A view of the inter­actions involving the hydrazinium cation is shown in Fig. 2 ▸ *a*. Each of the ammonium-N3—H atoms forms a charge-assisted hydrogen bond to a water mol­ecule, with the H*N*4 atom also forming a hydrogen bond to a nitro-O4 atom indicating that the H*N*4 atom is bifurcated [*i.e*.: N—H⋯(O,O)]. The amine-N4—H atoms form a hydrogen bond to a carboxyl­ate-O1 atom and to a water mol­ecule and at the same time accept a hydrogen bond from an amino-H atom, this being the only N—H⋯N hydrogen bond in the structure; the second amino-H atom forms an intra­molecular hydrogen bond with the carboxyl­ate-O1 atom, as mentioned above. Each of the water-H atoms forms a charge-assisted hydrogen bond with a carboxyl­ate-O atom, leading to a zigzag supra­molecular chain aligned along the *b* axis, as shown in Fig. 2 ▸ *b*. The chain comprises alternating twelve-membered {⋯OCO⋯HOH}_2_ and eight-membered {⋯O⋯HOH}_2_ synthons. As shown in Fig. 2 ▸ *c*, two of the ammonium-N3—H atoms bridge water mol­ecules in the chain shown in Fig. 2 ▸ *b* to form a non-symmetric, eight-membered {⋯HNH⋯OH⋯O⋯HO} synthon while the amine-H atoms provide a second bridge between water- and carboxyl­ate-O atoms to form a ten-membered {⋯HNH⋯OH⋯O⋯HOH⋯O} synthon. Further hydrogen bonds to water mol­ecules leads to the formation of additional synthons, *i.e*. ten-membered {⋯HNNH⋯O}_2_ and eight-membered {⋯HNH⋯O}_2_. A view of the unit-cell contents is shown in Fig. 2 ▸ *d*. In addition to the above, π(phen­yl)–π(phen­yl) inter­actions are noted between inversion-related rings with the inter-centroid separation being 3.6190 (8) Å [symmetry operation 1 − *x*, −*y*, 1 − *z*].

## Hirshfeld surface analysis   

**Figure 3:**
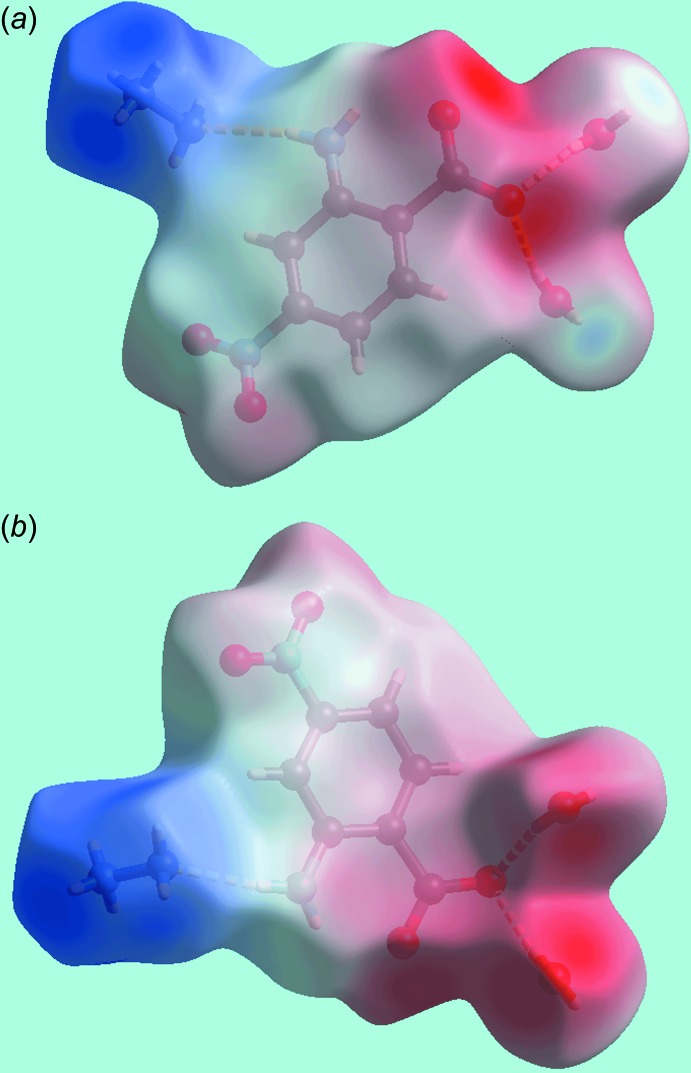
Two views of the Hirshfeld surface for (I) mapped over the electrostatic potential over the range −0.214 to +0.341 au; the red and blue regions represent negative and positive electrostatic potentials, respectively.

**Figure 4:**
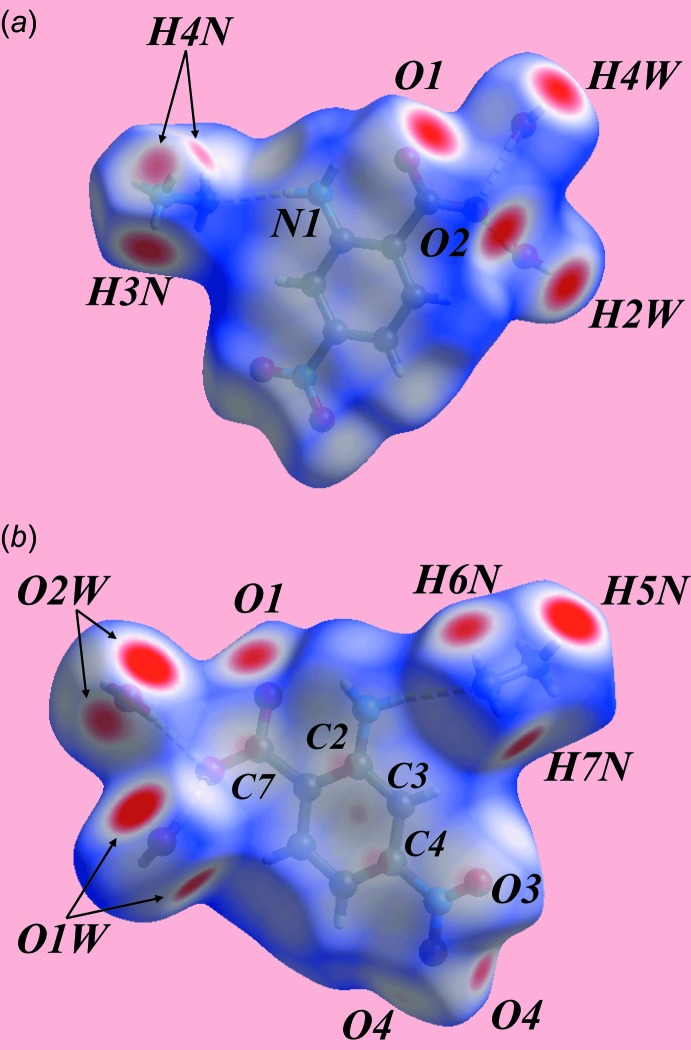
Two views of the Hirshfeld surface for (I) mapped over *d*_norm_ over the range −0.352 to 1.156 au.

The Hirshfeld surface analysis of (I) provides additional insight into its mol­ecular packing and was performed in accord with a recent study of related ammonium salts (Wardell *et al.*, 2016 ▸). The Hirshfeld surface mapped over electrostatic potential in Fig. 3 ▸ highlights the positive potential (blue region) around the hydrazinium cation and the negative potential (red) about the carboxyl­ate-oxygen atoms of the nitro­benzoate anion. The numerous bright-, diminutive- and faint-red spots appearing on the Hirshfeld surface mapped over *d*_norm_ in Fig. 4 ▸ are indicative of the variety of inter­molecular inter­actions in the crystal. The pair of charge-assisted water-O—H⋯O(carboxyl­ate) hydrogen bonds between the water-O—H2*W* and —H4*W* atoms and carboxyl­ate-O1 and -O2 atoms are evident through the bright-red spots appearing near the respective donor and acceptor atoms, Fig. 4 ▸ *a*. The donors of these inter­actions appear as light-blue spots near the water O—H atoms and the acceptors as red regions surrounding carboxyl­ate-O1 and -O2 atoms on the Hirshfeld surface mapped over electrostatic potential in Fig. 3 ▸.

**Figure 5:**
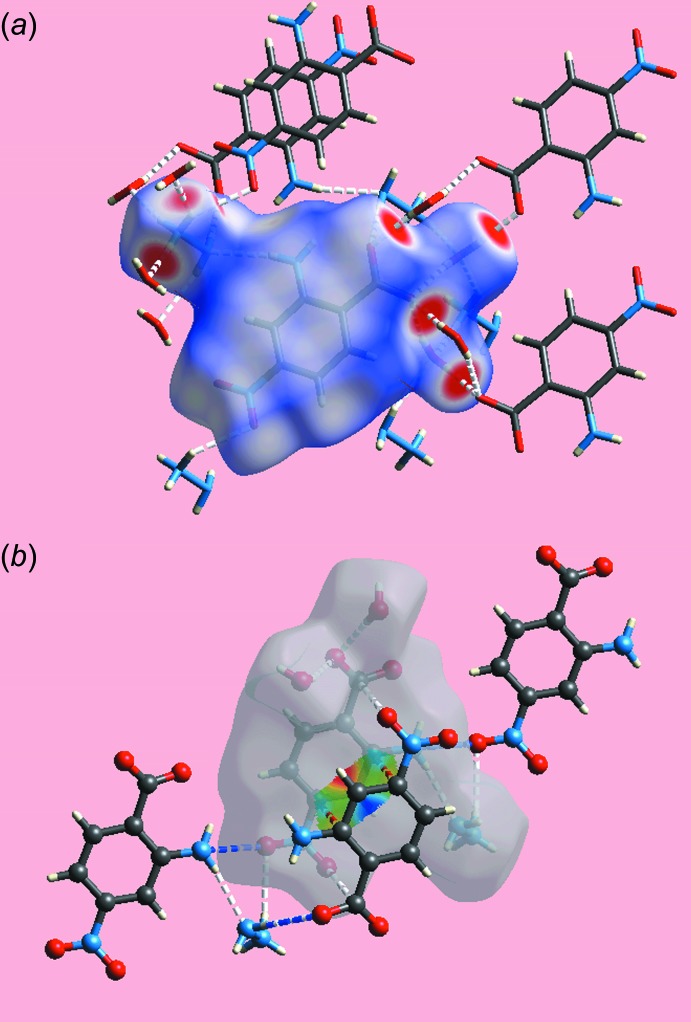
View of Hirshfeld surface for (I) mapped (*a*) over *d*_norm_ about a reference mol­ecule showing hydrogen bonds as white dashed lines and (*b*) mapped with the shape-index property about a reference ion-pair. The short inter­atomic C⋯C, N⋯O and C⋯O contacts are indicated with red, blue and white dotted lines, respectively

The two pairs of bright-red spots near each water-O1*W* and -O2*W* atoms, and near the hydrazinium-H3*N*, H4*N*, H5*N* and H7*N* atoms in Fig. 4 ▸ *b* are indicative of the hydrazinium-N—H⋯O(water) hydrogen bonds. In the same way, the amine-N4—H6*N*⋯O1 hydrogen bond is also viewed as a pair of bright-red spots near these atoms in Fig. 4 ▸ *b*. The bifurcated ammonium-H*N*4 atom, forming comparatively weaker N—H⋯O hydrogen bonds compared to those just described, is viewed as the diminutive red spot in Fig. 4 ▸ *a*. The presence of faint-red spots near the phenyl-C2–C4 atoms in Fig. 4 ▸ *b* indicate their participation in edge-to-edge overlap with a symmetry-related phenyl ring, as seen in the short inter­atomic C⋯C contacts listed in Table 2 ▸. In addition to above inter­molecular inter­actions, the crystal also features short inter­atomic C⋯O/O⋯C and N⋯O/O⋯N contacts, Table 2 ▸, which are viewed as very faint-red spots in Fig. 4 ▸. In Fig. 4 ▸ *b*, two spots are noted in the vicinity of the O4 atom, one corresponding to the conventional hydrogen bond and the other (to the left) to the weak H2*N*⋯O4 inter­action, Table 2 ▸. The immediate environments about a reference ion-pair within the *d*_norm_- and shape-index mapped Hirshfeld surfaces highlighting the O—H⋯O and O—H⋯N hydrogen bonds, and short inter­atomic C⋯C, C⋯O/O⋯C and N⋯O/O⋯N contacts are illustrated in Fig. 5 ▸ *a* and 5*b*, respectively.

**Figure 6:**
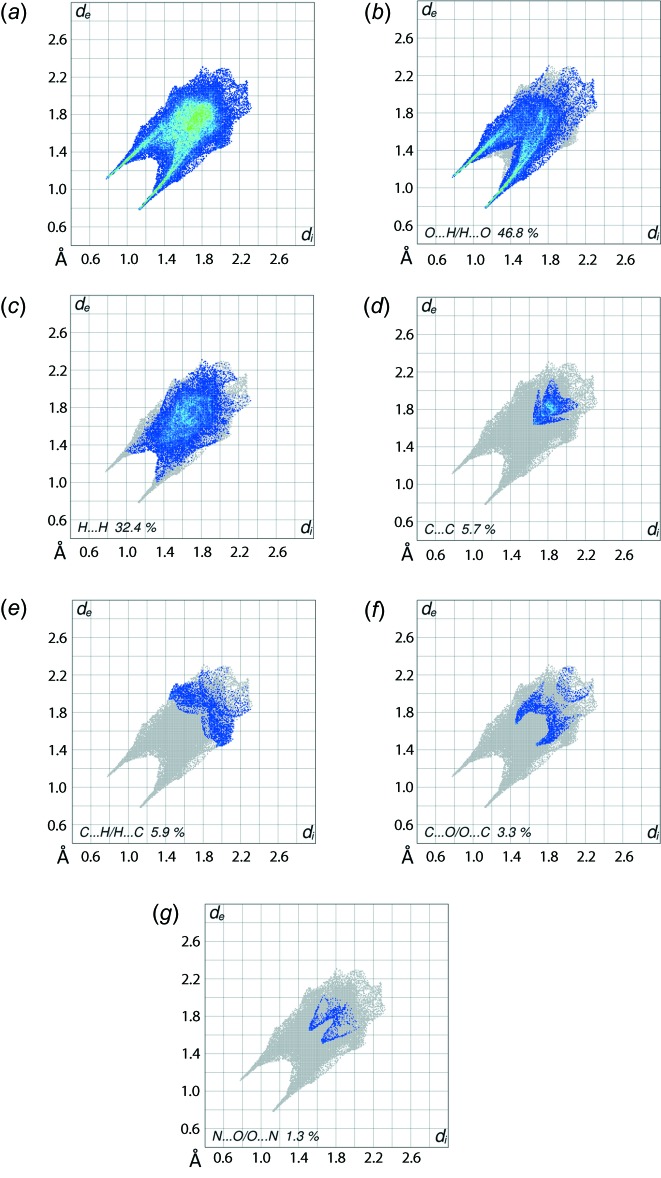
(*a*)The full two-dimensional fingerprint plot for (I) and fingerprint plots delineated into (*b*) O⋯H/H⋯O, (*c*) H⋯H, (*d*) C⋯C, (*e*) C⋯H/H⋯C, (*f*) C⋯O/O⋯C and (*g*) N⋯O/O⋯N contacts.

**Figure 7:**
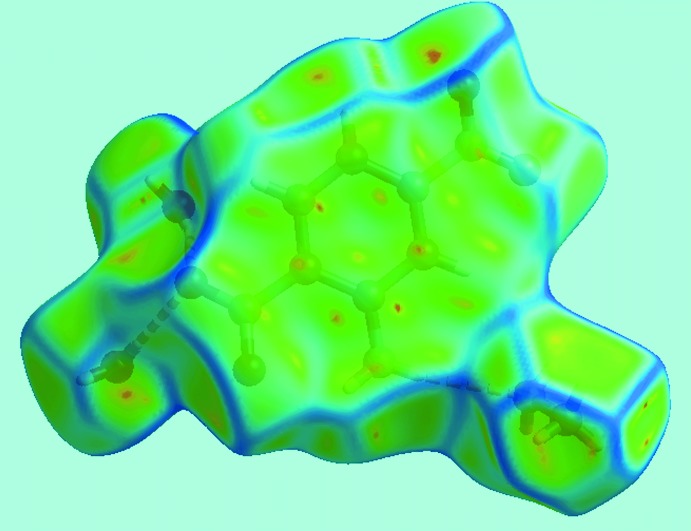
A view of Hirshfeld surfaces mapped over curvedness showing the flat region about the phenyl ring engaged in face-to-face π–π inter­actions.

The overall two-dimensional fingerprint plot for (I) and those delineated (McKinnon *et al.*, 2007 ▸) into O⋯H/H⋯O, H⋯H, C⋯C, C⋯H/H⋯C, C⋯O/O⋯C and N⋯O/O⋯N contacts are illustrated in Fig. 6 ▸ *a*–*g*, respectively; their relative contributions to the Hirshfeld surfaces are summarized in Table 3 ▸. It is important to note that the most significant contribution to the Hirshfeld surface in (I) comes from O⋯H/H⋯O contacts, *i.e*. 46.8%, due to the involvement of all the acidic hydrogen atoms in hydrogen bonds, mainly to oxygen, many of which are charge-assisted. Reflecting this dominance, sharp spikes are evident in the fingerprint plot delineated into O⋯H/H⋯O contacts shown in Fig. 6 ▸ *b.* The pair of green spikes have their tips at *d*_e_ + *d*_i_ ∼1.9 Å and extend linearly up to *d*_e_ + *d*_i_ ∼2.3 Å. The points merged within the plot up to *d*_e_ + *d*_i_ ∼2.7 Å indicate the presence of short inter­atomic O⋯H/H⋯O contacts, Table 2 ▸. The extensive hydrogen bonding is the cause of the relatively small percentage contribution to the Hirshfeld surface from H⋯H contacts, *i.e*. 32.4%, Fig. 6 ▸ *c*, as relatively few hydrogen atoms are available to form inter­atomic contacts. The pair of tips at *d*_e_ + *d*_i_ ∼2.3 Å in the mirror-reflected saw-tooth distribution are due to short inter­atomic H⋯H contacts involving water- and hydrazinium-hydrogen atoms, Table 2 ▸. The distributions of points in the fingerprint plot delineated into C⋯C contacts, shown in Fig. 6 ▸ *d*, represents two π–π stacking inter­actions. In the first of these, the symmetry-related phenyl rings have a face-to-face overlap to give the arrow-like distribution in lower (*d*_e_, *d*_i_) region at around *d*_e_ = *d*_i_ = 1.6 Å. This inter­action is also seen as the flat region appearing about the phenyl ring on the Hirshfeld surface mapped over curvedness, shown in Fig. 7 ▸. The other π–π stacking inter­action involves edge-to-edge overlap through short inter­atomic C⋯C contacts involving the C2–C4 atoms, Fig. 4 ▸ *b* and Table 2 ▸, and is viewed as the arrow-like distribution of points around *d*_e_ = *d*_i_ = 1.8 Å, *i.e*. adjacent to first arrow-like distribution. Even though C⋯H/H⋯C contacts have a significant contribution to the Hirshfeld surface, *i.e*. 5.9%, as seen from the fingerprint plot in Fig. 6 ▸ *e*, the inter­atomic separations are much greater than sum of their van der Waals radii and hence do not appear to have influence on the mol­ecular packing. The presence of short inter­atomic C⋯O/O⋯C and N⋯O/O⋯N contacts in the crystal, Table 2 ▸, is also evident from the small but significant contributions of 3.3 and 1.3%, respectively, to the Hirshfeld surfaces and appear as pairs of forceps-like tips, Fig. 6 ▸ *f*, and conical tips, Fig. 6 ▸ *g*, at *d*_e_ + *d*_i_ ∼3.1 Å in their respective fingerprint plots. The small contributions from the other inter­atomic O⋯O, C⋯N/N⋯C, N⋯N and N⋯H/H⋯N contacts listed in Table 2 ▸ have a negligible effect on the packing in the crystal.

## Database survey   

In the *Chemical context* section above, it was indicated that in the crystallographic literature there are several ammonium salts of 2-amino-4-nitro­benzoate anions. The ammonium cations range from the simple ammonium cation (Smith, 2014*b* ▸) to *R*_2_NH_2_, *i.e. R* = Me, *n*-Bu (Wardell *et al.*, 2016 ▸), Cy (Smith *et al.*, 2004 ▸) and R_2_ = (CH_2_CH_2_)_2_O (Smith & Lynch, 2016 ▸). More exotic examples of ammonium cations are found with [(H_2_N)_2_C=NH_2_]^+^, *i.e*. guanidinium (Smith *et al.*, 2007 ▸) and the dication, [H_3_NCH_2_CH_2_NH_3_]^2+^ (Smith *et al.*, 2002 ▸). Key geometric data for these are collated in Table 4 ▸. From these data it is apparent that the dihedral angle formed between the the carboxyl­ate group and benzene ring in (I) is at the upper end of structures included in Table 4 ▸, and in the same way, the angle between the nitro group and benzene ring is in the upper range of comparable angles. Given that the relationship between the carboxyl­ate and nitro groups in (I) is conrotatory, the dihedral angle between these groups in (I), at 26.73 (14)°, is the greatest among the series.

## Synthesis and crystallization   

Solutions of 2-amino-4-nitrobenzoic acid (1 mmol) in MeOH (10 ml) and hydrazine (1 mmol) in MeOH (15 ml) were mixed and heated under reflux for 30 min. The reaction mixture was left at room temperatures for three days and the red blocks that formed were collected. M.p. 375–377 K (dec.). IR (KBr: cm^−1^): 3514(*s*), 3399(*s*), 3400–2500(*br*,*s*), 1680, 1553, 1526, 1425, 1359, 1276, 830, 733.

## Refinement   

Crystal data, data collection and structure refinement details are summarized in Table 5 ▸. Carbon-bound H-atoms were placed in calculated positions (C—H = 0.95–0.99 Å) and were included in the refinement in the riding-model approximation, with *U*_iso_(H) set to 1.2*U*_eq_(C). The O- and N-bound H atoms were located from difference maps, but refined with O—H = 0.84±0.01 Å and *U*_iso_(H) = 1.5*U*_eq_(O), and with N—H = 0.86–0.88±0.01 Å and *U*_iso_(H) = 1.2*U*_eq_(N), respectively. Owing to poor agreement, two reflections, *i.e*. (202) and (212), were omitted from the final cycles of refinement.

## Supplementary Material

Crystal structure: contains datablock(s) I, global. DOI: 10.1107/S2056989017004352/hb7667sup1.cif

Structure factors: contains datablock(s) I. DOI: 10.1107/S2056989017004352/hb7667Isup2.hkl

CCDC reference: 1538892

Additional supporting information:  crystallographic information; 3D view; checkCIF report

## Figures and Tables

**Table 1:** Hydrogen-bond geometry (Å, °)

*D*—H⋯*A*	*D*—H	H⋯*A*	*D*⋯*A*	*D*—H⋯*A*
N1—H1*N*⋯O1	0.88 (1)	2.07 (1)	2.7098 (15)	129 (1)
N1—H2*N*⋯N4	0.88 (1)	2.29 (1)	3.1403 (16)	165 (1)
N3—H3*N*⋯O1*W* ^i^	0.86 (1)	1.97 (1)	2.8136 (15)	167 (1)
N3—H4*N*⋯O2*W* ^i^	0.87 (2)	2.25 (2)	2.8969 (16)	132 (1)
N3—H4*N*⋯O4^ii^	0.87 (2)	2.34 (1)	3.0739 (15)	143 (1)
N3—H5*N*⋯O2*W* ^iii^	0.87 (1)	1.93 (2)	2.7862 (15)	167 (1)
N4—H6*N*⋯O1^iii^	0.87 (1)	2.20 (1)	3.0623 (15)	178 (1)
N4—H7*N*⋯O1*W* ^iv^	0.87 (1)	2.20 (1)	3.0106 (15)	155 (1)
O1*W*—H1*W*⋯O2	0.86 (2)	1.97 (2)	2.8071 (14)	166 (2)
O1*W*—H2*W*⋯O2^v^	0.83 (2)	1.90 (2)	2.7208 (13)	171 (2)
O2*W*—H3*W*⋯O2	0.85 (2)	1.92 (2)	2.7479 (13)	165 (2)
O2*W*—H4*W*⋯O1^vi^	0.85 (2)	1.91 (2)	2.7627 (14)	175 (2)

Symmetry codes: (i) 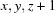; (ii) 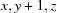; (iii) 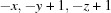; (iv) 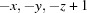; (v) 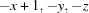; (vi) 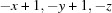.

**Table 2:** Summary of short inter­atomic contacts (Å) in (I)

Contact	Distance	Symmetry operation
H5*N*⋯H3*W*	2.38 (2)	-*x*, 1 − *y*, 1 − *z*
H7*N*⋯H2*W*	2.34 (2)	-*x*, −*y*, 1 − *z*
H4*N*⋯O3	2.626 (15)	*x*, 1 + *y*, *z*
H5⋯O4	2.61	1 − *x*, −1 − *y*, 1 − *z*
H2*N*⋯O4	2.652 (15)	*x*, 1 + *y*, *z*
N1⋯O4	3.0205 (15)	*x*, 1 + *y*, *z*
C7⋯O3	3.1231 (17)	-*x*, −*y*, 1 − *z*
C2⋯C4	3.2936 (19)	-*x*, −*y*, 1 − *z*
C3⋯C3	3.3235 (19)	-*x*, −*y*, 1 − *z*

**Table 3:** Percentage contribution to inter­atomic contacts from the Hirshfeld surface for (I)

Contact	Percentage contribution
O⋯H/H⋯O	46.8
H⋯H	32.4
C⋯H/H⋯C	5.9
C⋯C	5.7
C⋯O/O⋯C	3.3
O⋯O	1.6
N⋯O/O⋯N	1.3
C⋯N / N⋯C	1.2
N⋯N	1.0
N⋯H/H⋯N	0.8

**Table 4:** Geometric data (°) for ammonium salts of 2-amino-4-nitro­benzoate. Extreme values for each parameter are bolded

cation	*Z*′	C_6_/CO_2_	C_6_/NO_2_	CO_2_/NO_2_	Ref.
[NH_4_]^+^ *^*a*^*	1	**26.4 (3)**	2.9 (3)	24.1 (4)	Smith (2014*b* ▸)
[Me_2_NH_2_]^+^	1	11.45 (13)	3.71 (15)	7.9 (2)	Wardell *et al.* (2016 ▸)
[*n*-Bu_2_NH_2_]^+^	2	12.73 (6)	4.30 (10)	17.02 (8)	Wardell *et al.* (2016 ▸)
		8.1 (4)	**12.6 (3)**	19.0 (5)	
[Cy_2_NH_2_]^+^	2	9.87 (10)	7.58 (15)	3.42 (19)	Smith *et al.* (2004 ▸)
		9.52 (9)	7.86 (11)	3.92 (2)	
[O(CH_2_CH_2_)_2_NH_2_]^+^	1	17.92 (9)	1.28 (11)	19.19 (13)	Smith & Lynch (2016 ▸)
[(H_2_N)_2_C=NH_2_]^+^ *^*a*^*	1	5.88 (11)	5.64 (12)		Smith *et al.* (2007 ▸)
[H_3_NCH_2_CH_2_NH_3_]^2+^ *^*b*^*	1	**3.44 (14)**	**0.69 (11)**	**3.2 (2)**	Smith *et al.* (2002 ▸)
[H_2_NNH_3_]^2+^ *^*b*^*	1	18.80 (10)	8.04 (9)	**26.73 (14)**	this work

Notes: (*a*) crystallized as a monohydrate; (*b*) crystallized as a dihydrate.

**Table 5:** Experimental details

Crystal data	
Chemical formula	H_5_N_2_^+^·C_7_H_5_N_2_O_4_^−^·2H_2_O
*M*_r_	250.22
Crystal system, space group	Triclinic, *P* 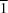
Temperature (K)	120
*a*, *b*, *c* (Å)	6.9695 (2), 8.0960 (3), 10.5316 (3)
α, β, γ (°)	76.468 (2), 73.251 (2), 75.390 (2)
*V* (Å^3^)	542.23 (3)
*Z*	2
Radiation type	Mo *K*α
μ (mm^−1^)	0.13
Crystal size (mm)	0.41 × 0.22 × 0.13

Data collection	
Diffractometer	Bruker–Nonius Roper CCD camera on κ-goniostat
Absorption correction	Multi-scan (*SADABS*; Sheldrick, 2007 ▸)
*T*_min_, *T*_max_	0.644, 0.746
No. of measured, independent and observed [*I* > 2σ(*I*)] reflections	11539, 2497, 2147
*R*_int_	0.034

Refinement	
*R*[*F* ^2^ > 2σ(*F* ^2^)], *wR*(*F* ^2^), *S*	0.039, 0.110, 1.05
No. of reflections	2497
No. of parameters	187
No. of restraints	13
Δρ_max_, Δρ_min_ (e Å^−3^)	0.29, −0.30

Computer programs: *DENZO* (Otwinowski & Minor, 1997 ▸), *COLLECT* (Hooft, 1998 ▸), *SHELXS97* (Sheldrick, 2008 ▸), *SHELXL2014* (Sheldrick, 2015 ▸), *ORTEP-3 for Windows* (Farrugia, 2012 ▸) and *DIAMOND* (Brandenburg, 2006 ▸) and *publCIF* (Westrip, 2010 ▸).

## supplementary crystallographic information

### Crystal data

**Table:**

H_5_N_2_^+^·C_7_H_5_N_2_O_4_^−^·2H_2_O	*Z* = 2
*M**_r_* = 250.22	*F*(000) = 264
Triclinic, *P*1	*D*_x_ = 1.533 Mg m^−^^3^
*a* = 6.9695 (2) Å	Mo *K*α radiation, λ = 0.71073 Å
*b* = 8.0960 (3) Å	Cell parameters from 7476 reflections
*c* = 10.5316 (3) Å	θ = 2.9–27.5°
α = 76.468 (2)°	µ = 0.13 mm^−^^1^
β = 73.251 (2)°	*T* = 120 K
γ = 75.390 (2)°	Block, red
*V* = 542.23 (3) Å^3^	0.41 × 0.22 × 0.13 mm

### Data collection

**Table:**

Bruker–Nonius Roper CCD camera on κ-goniostat diffractometer	2497 independent reflections
Radiation source: Bruker–Nonius FR591 rotating anode	2147 reflections with *I* > 2σ(*I*)
Graphite monochromator	*R*_int_ = 0.034
Detector resolution: 9.091 pixels mm^-1^	θ_max_ = 27.6°, θ_min_ = 3.1°
φ & ω scans	*h* = −9→8
Absorption correction: multi-scan (SADABS; Sheldrick, 2007)	*k* = −10→10
*T*_min_ = 0.644, *T*_max_ = 0.746	*l* = −13→13
11539 measured reflections	

### Refinement

**Table:**

Refinement on *F*^2^	13 restraints
Least-squares matrix: full	Hydrogen site location: mixed
*R*[*F*^2^ > 2σ(*F*^2^)] = 0.039	*w* = 1/[σ^2^(*F*_o_^2^) + (0.0646*P*)^2^ + 0.1366*P*] where *P* = (*F*_o_^2^ + 2*F*_c_^2^)/3
*wR*(*F*^2^) = 0.110	(Δ/σ)_max_ < 0.001
*S* = 1.05	Δρ_max_ = 0.29 e Å^−^^3^
2497 reflections	Δρ_min_ = −0.30 e Å^−^^3^
187 parameters	

### Special details

**Table:**

Geometry. All esds (except the esd in the dihedral angle between two l.s. planes) are estimated using the full covariance matrix. The cell esds are taken into account individually in the estimation of esds in distances, angles and torsion angles; correlations between esds in cell parameters are only used when they are defined by crystal symmetry. An approximate (isotropic) treatment of cell esds is used for estimating esds involving l.s. planes.

### Fractional atomic coordinates and isotropic or equivalent isotropic displacement parameters (Å^2^)

**Table:**

	*x*	*y*	*z*	*U*_iso_*/*U*_eq_	
O1	0.40595 (14)	0.36669 (11)	0.25616 (9)	0.0168 (2)	
O2	0.46220 (13)	0.18017 (11)	0.11781 (9)	0.0164 (2)	
O3	0.04781 (14)	−0.28112 (12)	0.77375 (9)	0.0224 (2)	
O4	0.24000 (15)	−0.47250 (11)	0.65475 (9)	0.0195 (2)	
N1	0.15996 (17)	0.28747 (14)	0.50370 (11)	0.0163 (2)	
H1N	0.218 (2)	0.3697 (17)	0.4483 (14)	0.020*	
H2N	0.107 (2)	0.301 (2)	0.5877 (10)	0.020*	
N2	0.17056 (16)	−0.32135 (13)	0.67034 (10)	0.0150 (2)	
C1	0.34738 (18)	0.08075 (15)	0.35268 (12)	0.0128 (3)	
C2	0.23062 (17)	0.12171 (15)	0.47960 (12)	0.0123 (3)	
C3	0.17447 (18)	−0.01538 (16)	0.58371 (12)	0.0133 (3)	
H3	0.0949	0.0079	0.6698	0.016*	
C4	0.23572 (18)	−0.18300 (15)	0.55975 (12)	0.0132 (3)	
C5	0.35262 (19)	−0.22781 (16)	0.43707 (13)	0.0152 (3)	
H5	0.3943	−0.3450	0.4241	0.018*	
C6	0.40531 (19)	−0.09256 (16)	0.33461 (12)	0.0148 (3)	
H6	0.4836	−0.1183	0.2489	0.018*	
C7	0.40929 (18)	0.21917 (15)	0.23517 (12)	0.0130 (3)	
N3	−0.00982 (17)	0.32666 (14)	0.90634 (11)	0.0161 (2)	
H3N	0.045 (2)	0.2268 (14)	0.9450 (14)	0.019*	
H4N	0.086 (2)	0.3844 (19)	0.8652 (14)	0.019*	
H5N	−0.102 (2)	0.3825 (19)	0.9655 (13)	0.019*	
N4	−0.09239 (17)	0.30241 (15)	0.80197 (11)	0.0180 (3)	
H6N	−0.179 (2)	0.3973 (15)	0.7857 (16)	0.022*	
H7N	−0.159 (2)	0.2192 (17)	0.8375 (15)	0.022*	
O1W	0.21973 (14)	0.02486 (12)	0.02803 (10)	0.0184 (2)	
H1W	0.279 (3)	0.070 (2)	0.0681 (16)	0.028*	
H2W	0.312 (2)	−0.033 (2)	−0.0235 (15)	0.028*	
O2W	0.31998 (14)	0.45790 (12)	−0.06089 (9)	0.0170 (2)	
H3W	0.381 (2)	0.382 (2)	−0.0080 (15)	0.025*	
H4W	0.409 (2)	0.506 (2)	−0.1221 (15)	0.025*	

### Atomic displacement parameters (Å^2^)

**Table:**

	*U*^11^	*U*^22^	*U*^33^	*U*^12^	*U*^13^	*U*^23^
O1	0.0211 (5)	0.0127 (5)	0.0162 (4)	−0.0063 (3)	−0.0025 (3)	−0.0008 (3)
O2	0.0197 (5)	0.0145 (5)	0.0126 (4)	−0.0027 (3)	−0.0020 (3)	−0.0008 (3)
O3	0.0216 (5)	0.0207 (5)	0.0165 (5)	−0.0019 (4)	0.0023 (4)	0.0019 (4)
O4	0.0268 (5)	0.0115 (5)	0.0204 (5)	−0.0057 (4)	−0.0067 (4)	0.0005 (3)
N1	0.0209 (6)	0.0119 (5)	0.0145 (5)	−0.0046 (4)	−0.0005 (4)	−0.0026 (4)
N2	0.0151 (5)	0.0145 (5)	0.0158 (5)	−0.0045 (4)	−0.0059 (4)	0.0009 (4)
C1	0.0127 (6)	0.0121 (6)	0.0139 (6)	−0.0033 (4)	−0.0043 (4)	−0.0004 (4)
C2	0.0113 (6)	0.0120 (6)	0.0147 (6)	−0.0027 (4)	−0.0058 (4)	−0.0009 (4)
C3	0.0122 (6)	0.0148 (6)	0.0126 (6)	−0.0031 (4)	−0.0033 (4)	−0.0012 (4)
C4	0.0129 (6)	0.0127 (6)	0.0140 (6)	−0.0046 (4)	−0.0052 (4)	0.0021 (5)
C5	0.0162 (6)	0.0102 (6)	0.0187 (6)	−0.0014 (4)	−0.0055 (5)	−0.0014 (5)
C6	0.0146 (6)	0.0148 (6)	0.0140 (6)	−0.0025 (4)	−0.0024 (4)	−0.0027 (5)
C7	0.0109 (6)	0.0129 (6)	0.0142 (6)	−0.0016 (4)	−0.0035 (4)	−0.0005 (4)
N3	0.0162 (5)	0.0139 (5)	0.0173 (6)	−0.0033 (4)	−0.0034 (4)	−0.0017 (4)
N4	0.0207 (6)	0.0166 (6)	0.0173 (6)	−0.0050 (4)	−0.0047 (4)	−0.0025 (4)
O1W	0.0177 (5)	0.0171 (5)	0.0203 (5)	−0.0020 (4)	−0.0038 (4)	−0.0056 (4)
O2W	0.0154 (4)	0.0169 (5)	0.0161 (5)	−0.0045 (3)	−0.0037 (3)	0.0029 (4)

### Geometric parameters (Å, º)

**Table:**

O1—C7	1.2579 (15)	C4—C5	1.3886 (18)
O2—C7	1.2746 (15)	C5—C6	1.3855 (18)
O3—N2	1.2299 (14)	C5—H5	0.9500
O4—N2	1.2280 (14)	C6—H6	0.9500
N1—C2	1.3644 (16)	N3—N4	1.4492 (15)
N1—H1N	0.873 (9)	N3—H3N	0.864 (9)
N1—H2N	0.877 (9)	N3—H4N	0.865 (9)
N2—C4	1.4692 (15)	N3—H5N	0.872 (9)
C1—C6	1.4010 (17)	N4—H6N	0.864 (9)
C1—C2	1.4153 (17)	N4—H7N	0.865 (9)
C1—C7	1.5035 (16)	O1W—H1W	0.856 (14)
C2—C3	1.4106 (17)	O1W—H2W	0.834 (13)
C3—C4	1.3760 (18)	O2W—H3W	0.847 (13)
C3—H3	0.9500	O2W—H4W	0.852 (13)
			
C2—N1—H1N	118.5 (11)	C6—C5—H5	121.8
C2—N1—H2N	117.4 (10)	C4—C5—H5	121.8
H1N—N1—H2N	117.3 (15)	C5—C6—C1	122.44 (12)
O4—N2—O3	122.87 (10)	C5—C6—H6	118.8
O4—N2—C4	118.34 (10)	C1—C6—H6	118.8
O3—N2—C4	118.79 (10)	O1—C7—O2	123.11 (11)
C6—C1—C2	119.62 (11)	O1—C7—C1	119.32 (11)
C6—C1—C7	118.73 (11)	O2—C7—C1	117.57 (11)
C2—C1—C7	121.65 (11)	N4—N3—H3N	109.2 (10)
N1—C2—C3	118.52 (11)	N4—N3—H4N	105.8 (10)
N1—C2—C1	123.24 (11)	H3N—N3—H4N	108.1 (15)
C3—C2—C1	118.13 (11)	N4—N3—H5N	112.3 (10)
C4—C3—C2	119.56 (11)	H3N—N3—H5N	110.6 (15)
C4—C3—H3	120.2	H4N—N3—H5N	110.6 (15)
C2—C3—H3	120.2	N3—N4—H6N	105.4 (11)
C3—C4—C5	123.75 (11)	N3—N4—H7N	106.2 (11)
C3—C4—N2	117.70 (11)	H6N—N4—H7N	108.5 (16)
C5—C4—N2	118.54 (11)	H1W—O1W—H2W	106.4 (15)
C6—C5—C4	116.48 (11)	H3W—O2W—H4W	108.3 (15)
			
C6—C1—C2—N1	−176.80 (11)	O3—N2—C4—C5	−171.63 (11)
C7—C1—C2—N1	2.39 (18)	C3—C4—C5—C6	−0.98 (18)
C6—C1—C2—C3	−0.76 (17)	N2—C4—C5—C6	178.13 (10)
C7—C1—C2—C3	178.43 (10)	C4—C5—C6—C1	0.86 (18)
N1—C2—C3—C4	176.90 (11)	C2—C1—C6—C5	−0.02 (18)
C1—C2—C3—C4	0.67 (17)	C7—C1—C6—C5	−179.23 (11)
C2—C3—C4—C5	0.22 (18)	C6—C1—C7—O1	−161.98 (11)
C2—C3—C4—N2	−178.90 (10)	C2—C1—C7—O1	18.83 (17)
O4—N2—C4—C3	−173.12 (10)	C6—C1—C7—O2	18.36 (16)
O3—N2—C4—C3	7.53 (16)	C2—C1—C7—O2	−160.84 (11)
O4—N2—C4—C5	7.72 (16)		

### Hydrogen-bond geometry (Å, º)

**Table:**

*D*—H···*A*	*D*—H	H···*A*	*D*···*A*	*D*—H···*A*
N1—H1*N*···O1	0.88 (1)	2.07 (1)	2.7098 (15)	129 (1)
N1—H2*N*···N4	0.88 (1)	2.29 (1)	3.1403 (16)	165 (1)
N3—H3*N*···O1*W*^i^	0.86 (1)	1.97 (1)	2.8136 (15)	167 (1)
N3—H4*N*···O2*W*^i^	0.87 (2)	2.25 (2)	2.8969 (16)	132 (1)
N3—H4*N*···O4^ii^	0.87 (2)	2.34 (1)	3.0739 (15)	143 (1)
N3—H5*N*···O2*W*^iii^	0.87 (1)	1.93 (2)	2.7862 (15)	167 (1)
N4—H6*N*···O1^iii^	0.87 (1)	2.20 (1)	3.0623 (15)	178 (1)
N4—H7*N*···O1*W*^iv^	0.87 (1)	2.20 (1)	3.0106 (15)	155 (1)
O1*W*—H1*W*···O2	0.86 (2)	1.97 (2)	2.8071 (14)	166 (2)
O1*W*—H2*W*···O2^v^	0.83 (2)	1.90 (2)	2.7208 (13)	171 (2)
O2*W*—H3*W*···O2	0.85 (2)	1.92 (2)	2.7479 (13)	165 (2)
O2*W*—H4*W*···O1^vi^	0.85 (2)	1.91 (2)	2.7627 (14)	175 (2)

Symmetry codes: (i) *x*, *y*, *z*+1; (ii) *x*, *y*+1, *z*; (iii) −*x*, −*y*+1, −*z*+1; (iv) −*x*, −*y*, −*z*+1; (v) −*x*+1, −*y*, −*z*; (vi) −*x*+1, −*y*+1, −*z*.

## References

[bb1] Brandenburg, K. (2006). *DIAMOND*. Crystal Impact GbR, Bonn, Germany.

[bb2] Chen, H.-L. & Huang, C.-F. (2009). *Synth. React. Inorg., Met.-Org., Nano-Met. Chem* **39**, 533–536.

[bb3] Farrugia, L. J. (2012). *J. Appl. Cryst.* **45**, 849–854.

[bb4] Groom, C. R., Bruno, I. J., Lightfoot, M. P. & Ward, S. C. (2016). *Acta Cryst.* B**72**, 171–179. 10.1107/S2052520616003954 PMC4822653 27048719

[bb5] Hooft, R. W. W. (1998). *COLLECT*. Nonius BV, Delft, The Netherlands.

[bb6] McKinnon, J. J., Jayatilaka, D. & Spackman, M. A. (2007). *Chem. Commun*. pp. 3814–3816. 10.1039/b704980c 18217656

[bb7] Otwinowski, Z. & Minor, W. (1997). *Methods in Enzymology*, Vol. 276, *Macromolecular Crystallography*, Part A, edited by C. W. Carter Jr & R. M. Sweet, pp. 307–326. New York: Academic Press.

[bb8] Sheldrick, G. M. (2007). *SADABS*. University of Göttingen, Germany.

[bb9] Sheldrick, G. M. (2008). *Acta Cryst.* A**64**, 112–122. 10.1107/S0108767307043930 18156677

[bb10] Sheldrick, G. M. (2015). *Acta Cryst.* C**71**, 3–8.

[bb11] Smith, G. (2013). *Acta Cryst.* C**69**, 1472–1477. 10.1107/S0108270113028977 24311493

[bb12] Smith, G. (2014*a*). *Acta Cryst.* E**70**, m192–m193. 10.1107/S1600536814008861 PMC4011243 24860320

[bb13] Smith, G. (2014*b*). Private communication (refcode DOBPIV). CCDC, Cambridge, England.

[bb14] Smith, G. & Lynch, D. E. (2016). *Acta Cryst.* C**72**, 105–111. 10.1107/S2053229615024821 26846493

[bb15] Smith, G. & Wermuth, U. D. (2011). *Acta Cryst.* E**67**, m1047–m1048. 10.1107/S1600536811026614 PMC3212135 22090837

[bb16] Smith, G., Wermuth, U. D. & Healy, P. C. (2004). *Acta Cryst.* E**60**, o684–o686. 10.1107/S0108270104015057 15295198

[bb17] Smith, G., Wermuth, U. D., Healy, P. C. & White, J. M. (2007). *Acta Cryst.* E**63**, o7–o9.

[bb18] Smith, G., Wermuth, U. D. & White, J. M. (2002). *Acta Cryst.* E**58**, o1088–o1090.

[bb19] Wardell, J. L., Jotani, M. M. & Tiekink, E. R. T. (2016). *Acta Cryst.* E**72**, 1618–1627. 10.1107/S2056989016016492 PMC5095847 27840722

[bb20] Wardell, J. L. & Tiekink, E. R. T. (2011). *J. Chem. Crystallogr.* **41**, 1418–1424.

[bb21] Wardell, S. M. S. V. & Wardell, J. L. (2016). *J. Chem. Crystallogr.* **46**, 34–43.

[bb22] Westrip, S. P. (2010). *J. Appl. Cryst.* **43**, 920–925.

